# Prevalence of virulence genes among VRE genotypes and their association with clinical outcome

**DOI:** 10.1186/1471-2334-14-S3-P44

**Published:** 2014-05-27

**Authors:** Ramamurthi Arularasi Aberna, Kesani Prabhakar

**Affiliations:** 1 Rajah Muthiah Dental College and Hospital, Annamalai University, Annamalai Nagar, India; 2 Rajah Muthiah Medical College and Hospital, Annamalai University, Annamalai Nagar, India

## Background

The dramatic upsurge in the incidence of VRE worldwide had contributed serious concerns in early and specific diagnosis, infection control measures and treatment. There exists a paucity of information regarding VRE genotypes and their virulence genes contributing to disease severity in India. This study determines the prevalence of VRE and the role of four virulence genes in their clinical outcome.

## Methods

Enterococci were isolated from a total of 2500 clinical specimens and species identification was done based on conventional methods. VRE were confirmed by agar dilution (vancomycin and teicoplanin) and by the presence of *vanA/vanB* genes. The presence of *esp*, *agg*, *gelE* and *cylA* gene among VRE were detected by PCR.

## Results

VRE were isolated from 7.3% of clinical specimens. VR *E.faecalis* and VR *E.faecium* were primarily derived from UTI and BSI (56.7% and 43.3%) respectively. Among the VRE isolates, 80% of *vanA* genes were noted in *E.faecium* and 64% of *vanB* genes were observed in *E.faecalis*. Most of VR *E.faecalis* harboured 2 virulence genes (38.5%) and VR *E.faecium* harboured one virulence gene (62.5%). The ability to cause invasive infections increased with the number of virulence genes harboured by *E.faecalis*. An association was observed between the presence of virulence gene *esp*, *cylA* and *agg* with UTI, BSI and IAP infections respectively among the VRE isolates.

## Conclusion

Isolation of VRE harbouring *vanA* and *vanB* gene gains significance as they are transmissible to co infecting/ resident organisms and to implement infection control measures.

